# Prevalence of *APC* and *PTEN* Alterations in Urachal Cancer

**DOI:** 10.1007/s12253-020-00872-6

**Published:** 2020-08-04

**Authors:** Nikolett Nagy, Henning Reis, Boris Hadaschik, Christian Niedworok, Orsolya Módos, Attila Szendrői, Krisztina Bíró, Thomas Hager, Thomas Herold, Jason Ablat, Peter C. Black, Krzysztof Okon, Yuri Tolkach, Anita Csizmarik, Csilla Oláh, David Keresztes, Felix Bremmer, Nadine T. Gaisa, Joerg Kriegsmann, Ilona Kovalszky, András Kiss, József Tímár, Marcell A. Szász, Michael Rink, Margit Fisch, Péter Nyirády, Tibor Szarvas

**Affiliations:** 1 Department of Urology, Semmelweis University, Budapest, 1082 Hungary; 2 Institute of Pathology, West German Cancer Center, University of Duisburg-Essen, University Hospital Essen, 45147 Essen, Germany; 3 Department of Urology, West German Cancer Center, University of Duisburg-Essen, University Hospital Essen, 45147 Essen, Germany; 4 National Institute of Oncology, Budapest, 1122 Hungary; 5 Vancouver Prostate Centre, University of British Columbia, Vancouver, V6H 3Z6 Canada; 6 Department of Pathomorphology, Jagiellonian University, 30252 Cracow, Poland; 7 Institute of Pathology, University of Bonn, 53113 Bonn, Germany; 8 Institute of Pathology, University of Göttingen, 37073 Göttingen, Germany; 9 Institute of Pathology, RWTH Aachen University, 52074 Aachen, Germany; 10 Cytology and Molecular Diagnostics Trier, Center for Histology, 54296 Trier, Germany; 11 1st Institute of Pathology and Expreimental Cancer Research, Semmelweis University, Budapest, 1085 Hungary; 12 2nd Department of Pathology, Semmelweis University, Budapest, 1091 Hungary; 13 Cancer Center, Semmelweis University, Budapest, 1083 Hungary; 14 Department of Urology, University Medical Center Hamburg-Eppendorf, 20246 Hamburg, Germany

**Keywords:** Urachal cancer, Colorectal cancer, Molecular genetics, Mutation, APC, ß-catenin

## Abstract

Urachal carcinoma (UrC) is a rare tumor with remarkable histological and molecular similarities to colorectal cancer (CRC). Adenomatous polyposis coli (*APC)* is the most frequently affected gene in CRC, but the prevalence and significance of its alterations in UrC is poorly understood. In addition, loss of phosphatase and tensin homologue (PTEN) was shown to be associated with therapy resistance in CRC. Our primary aim was to assess specific genetic alterations including *APC* and *PTEN* in a large series of UrC samples in order to identify clinically significant genomic alterations. We analyzed a total of 40 UrC cases. Targeted 5-gene *(APC, PTEN, DICER1, PRKAR1A, TSHR, WRN)* panel sequencing was performed on the Illumina MiSeq platform (*n* = 34). In addition, ß-catenin (*n* = 38) and PTEN (*n* = 30) expressions were assessed by immunohistochemistry. *APC* and *PTEN* genes were affected in 15% (5/34) and 6% (2/34) of cases. Two of five *APC* alterations (p.Y1075*, p.K1199*) were truncating pathogenic mutations. One of the two *PTEN* variants was a pathogenic frameshift insertion (p.C211fs). In 29% (11/38) of samples, at least some weak nuclear ß-catenin immunostaining was detected and PTEN loss was observed in 20% (6/30) of samples. The low prevalence of *APC* mutations in UrC represents a characteristic difference to CRC. Based on *APC* and ß-catenin results, the Wnt pathway seems to be rarely affected in UrC. Considering the formerly described involvement of PTEN protein loss in anti-EGFR therapy-resistance its immunohistochemical testing may have therapeutic relevance.

## Introduction

The urachus is an extraperitoneal fibromuscular band that connects the bladder dome and the umbilicus. During fetal development, the urachus ensures the communication between the forming bladder and the allantois. After the fourth month of embryonic life, the urachus usually transforms into a fibromuscular strand (i.e. median umbilical ligament). In up to one third of adults, this obliteration can be incomplete resulting in microscopic urachal residues. Incomplete regression of the urachal structure may lead to various diseases including urachal cancer (UrC) [1, 2].

UrC is an extremely rare disease with an estimated annual incidence of one in one million adults. The majority of UrC present as adenocarcinomas (~90%) with mucinous, intestinal, signet ring, not otherwise specified (NOS) or mixed histology. Because of its hidden anatomical location, UrC is mostly detected in progressed stages when patients present with hematuria resulting from its invasion into the urinary bladder. At this advanced stage, the overall 5-year survival is only about 50% [3–5].

Although urachal and colorectal adenocarcinomas (CRC) are different types of cancer, they share remarkable histopathological and clinical similarities. Also, their immunophenotype shows overlapping staining characteristics with only a few exceptions [4]. In contrast to CRC, the molecular background of UrC is only poorly understood. Data on UrC’s mutational pattern further highlighted its similarity to CRC with overlapping mutational patterns such as in *TP53*, *KRAS*, *SMAD4* and *NRAS* [6–17]. On the other hand, later studies also identified some significant molecular differences between UrC and CRC [6–12, 14–18].

In CRC, the adenomatous polyposis coli (*APC*) tumor suppressor gene has a critical role in the initiation of tumorigenesis. *APC* mutations lead to abnormal ß-catenin accumulation in the nucleus. This nuclear ß-catenin gets in contact with a member of the TCF/LEF family and acts as a transcriptional regulator on specific proliferation-associated target genes. APC mutations occur in more than 80% of CRC [17]. As a consequence, nuclear ß-catenin expression can be observed in the generality of CRC making ß-catenin a well established diagnostic and prognostic biomarker in CRC [19, 20]. The occurrence and role of *APC*-alterations and ß-catenin-expression are less well established in UrC.

Phosphatase and tensin homolog (PTEN) is a leading negative regulator of the PI3K signaling pathway and therefore known as a tumorsupressor gene. Its inactivating genetic mutations occur in up to 10% of CRCs [17]. In addition to genetic loss, PTEN is frequently downregulated by epigenetic silencing, leading to loss of PTEN expression [21]. Loss of PTEN protein expression was shown to be associated with resistance to anti-epidermal growth factor receptor (EGFR) therapy [22], while this therapy demonstrated effectiveness in UrC [14].

Therefore, we aimed to analyze relevant genetic alterations including the *APC* and *PTEN* genes as well as to assess the tissue protein expressions of β-catenin and PTEN in UrC.

## Materials and Methods

### Clinical Samples

Formalin-fixed paraffin embedded (FFPE) tumor tissues from 40 urachal adenocarcinoma patients were retrospectively collected from nine academic centers. Clinicopathological and follow-up data were retrieved from cooperating institutions using a uniform datasheet. Histopathological evaluation was performed according to criteria also adopted by the World Health Organization [23]. The study was performed in compliance with the Declaration of Helsinki and the institutional ethics committee approved the study protocol (16–6902-BO).

### Sample Preparation and Targeted Next-Generation Sequencing

FFPE tumor tissue blocks were cut and 4 μm-thick slides were stained with hematoxylin and eosin. Tumor containing areas were marked by a pathologist (H.R.) and macrodissection was performed. Sample preparation and targeted next-generation sequencing were performed as previously described [7]. For targeted sequencing, a customized panel was used containing regions of interest*.* The customized panel contains exonic and flanking intronic regions from 6 genes (*APC*: exons 1–16, *DICER1*: exons 1–28, *PRKAR1A*: exons 1–11, *PTEN*: exons 1–9, *TSHR*: exons 1–10, *WRN*: exons 2–35). The regions were covered by a total of 108 amplicons. In all runs, an average coverage of approximately 5000x was obtained. Analyses were performed using CLC Biomedical Genomics Workbench.

### Immunohistochemistry (IHC)

The ß-catenin-IHC (Ref: 503–2264, Zytomed Systems, Berlin, Germany; pretreatment: CC1, 90 °C, 16 min; dilution: 1:1000, 24 min) analysis was performed on 38 FFPE tumor UrC tissues while PTEN-IHC (clone: 138G6, Cell Signaling Technology, Cambridge, UK; pretreatment: CC1, 90 °C, 32 min; dilution: 1:200, 24 min) analysis was done on 30 available FFPE tumor samples. For IHC assays, 3 μm thick FFPE sections were cut. IHC was performed on a Ventana Benchmark Ultra system (Ventana Medical Systems, Tucson, AZ, USA) and visualization was conducted using OptiView DAB System (Ventana Medical Systems).

### Statistical Analysis

Statistical analyses were conducted with SPSS software package (v24 Chicago, IL). Kaplan-Meier survival analyses with the log-rank test were used to evaluate the impact of selected variables on overall survival. Furthermore, univariable Cox regression analyses were done. *P* values <0.05 were accounted statistically significant.

## Results

### Cohort Characteristics and Primary Treatment

The median age of patients at diagnosis was 51.5 years (range 24–78). Twenty-three patients (58%) were male and 17 (42%) were female. The most frequent symptom of UrC was haematuria reported in 83% of patients. Other symptoms included abdominal pain (10%) and palpable tumor mass (3%) (Table 1).

**Table 1:** Clinicopathological profile of 40 UrC cohort included in this study

		*n* = 40	(%)
Sex	Male	23	58
Age (year)	Median (Range)	51.5 (24–78)	
Haematuria	Yes	25	83
	No	5	17
	n.a.	10	–
Abdominal pain	Yes	3	10
	No	26	90
	n.a.	11	–
Palpable tumor	Yes	1	3
	No	29	97
	n.a.	10	–
UrC type	Intestinal	19	48
	Mucinous	14	35
	NOS	4	10
	SRC	2	5
	Mixed	1	3
Calcification	Yes	4	11
	No	34	89
	n.a.	2	–
Sheldon staging	I	0	0
	II	1	3
	III	24	63
	IV	13	34
	n.a.	2	–
Mayo staging	I	10	28
	II	13	36
	III	4	11
	IV	9	25
	n.a.	4	–
LN status	LN+	9	23
	LN-/LNx	31	78
Distant metastasis	M+	11	28
	M−/Mx	29	73
LN/M status	LN/M+	14	*35*
	LN/M- / LN/Mx	26	*65*
Surgery	TURB	3	*9*
	Partial cystectomy	24	*69*
	Radical cystectomy	8	*23*
	n.a.	5	*–*
Umbilectomy	Yes	16	*52*
	No	15	*48*
	n.a.	9	*–*

*Abbrevations*: *NOS* not otherwise specified, *SRC* signet ring cell carcinoma, *LN* lymp node, *LN*+ positive lymph node status, *LN*- negative lymph node status, *LNx* unknown lymph node status, *M*+ positive distant metastatic status, *M*- negative distant metastatic status, *Mx* unknown distant metastatic status, *TURB* transurethral resection of bladder, *n.a.* not available

All UrC were adenocarcinomas (ADC) mostly of the intestinal subtype (48%) followed by the mucinous subtype (35%). Dominant signet ring cell features (>50% of tumor cells) were seen in 2 cases (5%) while calcification was observed in 4 cases (11%) (Table 1).

Based on its specific anatomical location and invasion pattern, UrC requires a unique stage classification. The most often used systems are the Sheldon and Mayo staging systems (23, 24). One patient was staged as Sheldon stage II (3%), 24 were stage III (63%) and 13 were stage IV (34%). According to the Mayo system 10 UrCs were stage I (28%), 13 stage II (36%), 4 stage III (11%) and 9 were stage IV (25%) tumors (Table 1).

In our cohort, one third of patients had lymph node positive or distant metastatic disease at initial diagnosis (14/40, 35%). Data on surgical treatment were available for 35 patients. Most patients (24/35, 69%) were treated with partial cystectomy followed by radical cystecomy (8/35, 23%), and transurethral resection (3/35, 9%), while removal of median umbilical ligament with umbilicus has been performed in 52% of cases (Table 1).

### Genomic Alterations in Urachal Cancer

Sample purification in 34 of 40 cases yielded appropriate amount and quality of DNA sample for sequencing analysis. We analyzed the alterations of *APC, DICER1, PRKAR1A, PTEN, TSHR* and *WRN* genes. The *APC* and *PTEN* genes were affected in 15% (5/34) and 6% (2/34) of cases. Two of the *APC* alterations (p.Y1075*; c.3225 T > G, p.K1199*; c.3595A > T) were truncating mutations resulting in a truncated and non-functional APC protein and were therefore considered as pathogenic, while other three *APC* alterations were annotated as benign variants (p.L2722V; c.8164C > G, p.L666V; c.1996 T > G, p.N862K; c.2586C > G) (Table 2). One of the two *PTEN* alterations was a frameshift insertion, leading to a dysfunctional protein (p.C211fs; c.631_632insG) and was classified as a pathogenic mutation, while the other *PTEN* alteration was considered as benign (p.R308C; c.922C > T) (Table 2). In addition, *DICER1* intronic alterations (c.4206 + 8_4206 + 9insTTGTG, COSM1666515) were found in 29% (10/34) of UrC cases.

**Table 2:** Detected genomic alterations in UrC

Gene	Protein change	Nucleotide change	*Prediction*
*APC*	p.L2722V	c.8164C > G	*Benign*
*APC*	p.L666V	c.1996 T > G	*Benign*
*APC*	p.Y1075*	c.3225 T > G	*Pathogenic*
*APC*	p.N862K	c.2586C > G	*Benign*
*APC*	p.K1199*	c.3595A > T	*Pathogenic*
*PTEN*	p.R308C	c.922C > T	*Benign*
*PTEN*	p.C211fs	c.631_632insG	*Pathogenic*

### ß-Catenin and PTEN Immunoexpression

**Fig. 1:**
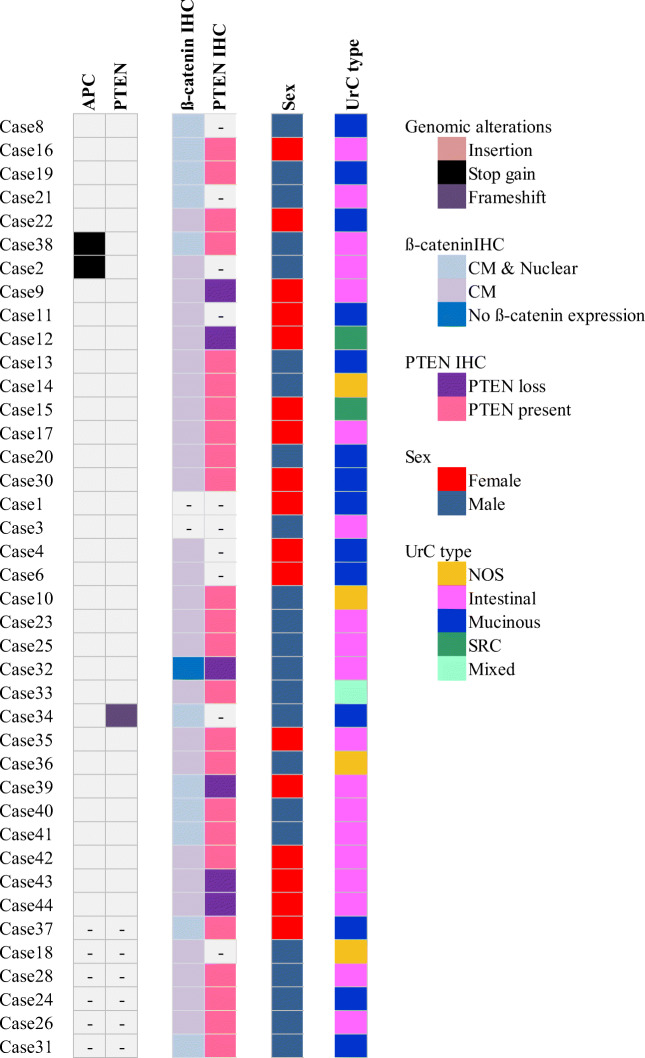
Comutation plot summarizing the genomic alterations for the 40 cases of UrC and mapping them to various clinicopathologic parameters. In case of APC and PTEN, only pathogenic mutations are shown. Abbrevations: IHC: immunhistochemistry, NOS: not otherwise specified, SRC: signet ring cell carcinoma, CM: cytoplasmic/membranous

**Fig. 2:**
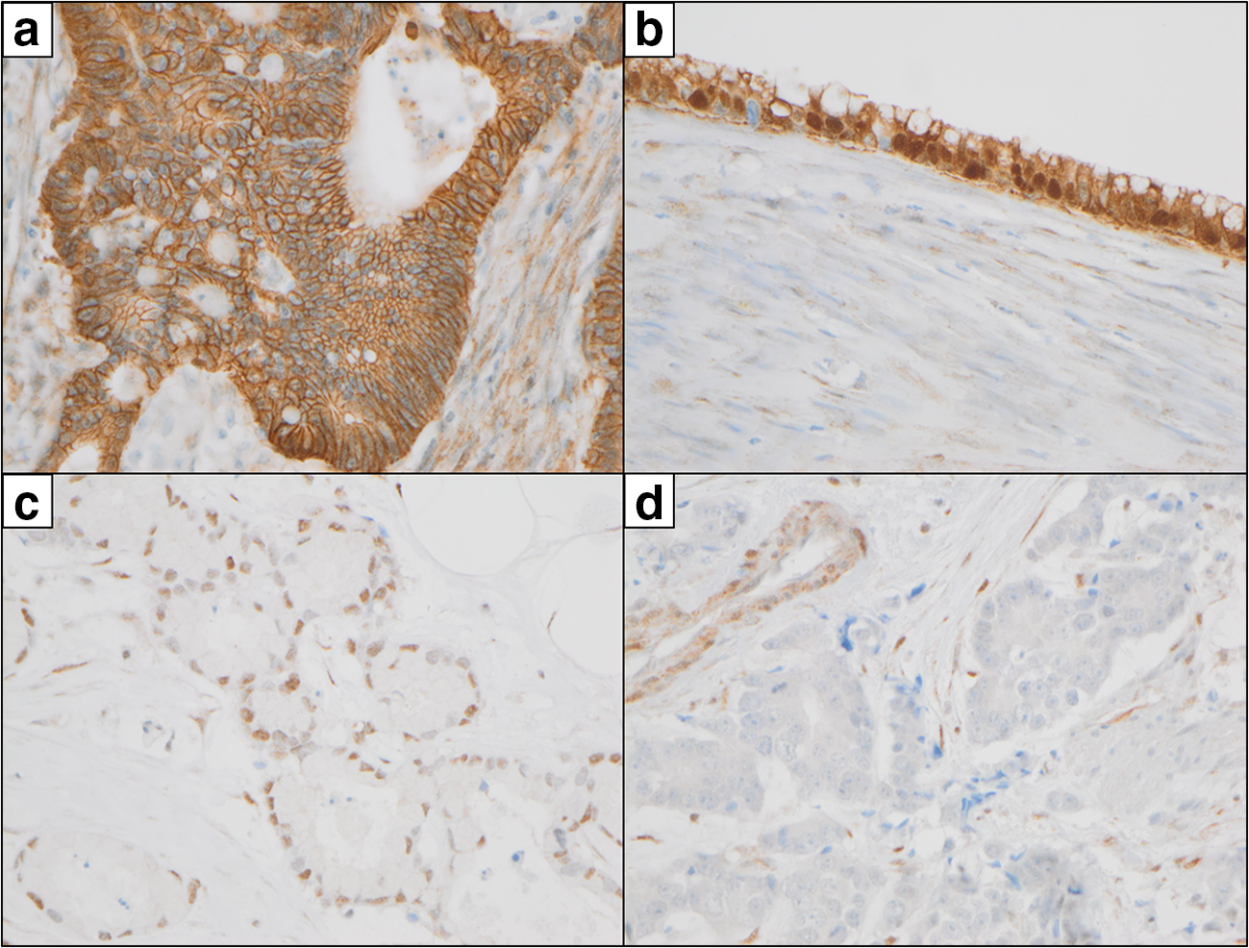
A typical example of strong membranous and cytoplasmic ß-catenin immunostaining in a case of UrC with intestinal differentiation in **a**) while in **b**) A case of mucionous UrC additionally showed strong nuclear ß-catenin immunopositivity of most cancer cell nuclei. In **c**) a case of UrC (NOS) with expression of PTEN is depicted while in **d**) another case of UrC (NOS) showed loss of PTEN-immunostaining with internal positive control. **a** 200x, **b**-**d**) 400x. Abbrevations: NOS: not otherwise specified

Immunohistochemistry for ß-catenin was performed in 38 cases. Positive membranous and cytoplasmic ß-catenin immunostaining was observed in 97% (37/38) of UrC samples. Any amount of clearly discernible nuclear immunostaining for ß-catenin was detected in 29% (11/38) of samples. In one of the two cases with truncating *APC* mutation (p.Y1075*; c.3225 T > G), nuclear expression of ß-catenin was observed. *PTEN* loss was observed in 6 of 30 (20%) UrC samples (Figs. 1 and 2).

### Survival Analysis

The median overall survival (OS) was 34 months (range: 2–212 months) and the median progression-free survival (PFS) was 22 months (range 0–117 months). Three-year and five-year OS was 71% and 64%, respectively. Postoperative progression was detected in 50% of cases (16/31). At the time of data evaluation, 65% of patient (22/34) were alive.

**Fig. 3:**
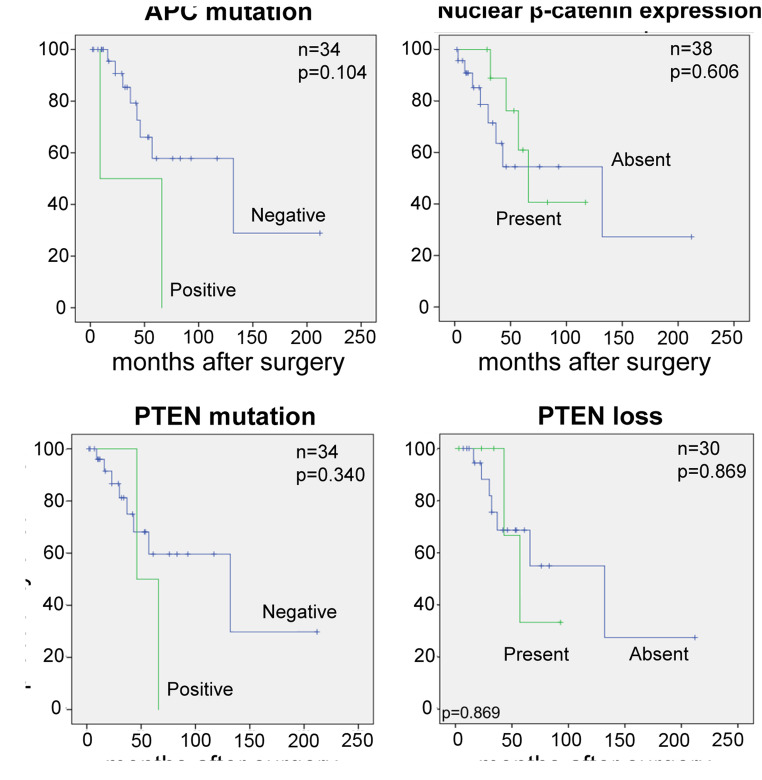
Kaplan-Meier curves of overall survival (OS) stratified by (**a**) APC mutation (**b**) nuclear ß-catenin expression (**c**) PTEN mutations, as well as (**d**) PTEN loss. Abbrevations: LN/M+: positive lymph node or distant metastatic status, LN/M-: negative lymph node and distant metastatic status, LN/Mx: unknown lymph node or distant metastatic status

Neither *APC* nor *PTEN* mutations were associated with OS, however these results must be interpreted with caution as only two cases demonstrated *APC* and *PTEN* mutations (Table 3) (Fig. 3a, c). Presence of nuclear ß-catenin and loss of PTEN expressions were not associated with shorter OS (*p* = 0.606 and *p* = 0.869) (Fig. 3b, d).

**Table 3:** Univariate analysis

Variables			Overall survival	
	n	HR	95% CI	P
Sheldon stage				
I-III	25	ref.		
IV	13	2.332	0.734–7.407	0.151
Mayo stage				
I-II	25	ref.		
III-IV	13	2.332	0.734–7.407	0.151
LN/M status				
LN/M- or LN/Mx	18	ref.		
LN/M+	13	1.982	0.564–6.969	0.286
PTEN mutations				
negative	32	ref.		
positive	2	2.124	0.436–10.337	0.351
PTEN staining				
present	24	ref.		
absent	6	1.144	0.230–5.687	0.869
APC status				
No mutation	32	ref.		
Mutation	2	3.464	0.704–17.056	0.127
β-Catenin nuclear staining				
negative	27	ref.		
positive	11	0.722	0.209–2.500	0.607

*Abbrevations*: *LN/M*+ positive lymph node or distant metastasis status, *LN/M*- negative lymph node and distant metastasis status, *LN/Mx* unknown lymph node or distant metastasis status

## Discussion

It is well known, that mutations of the *APC* tumor suppressor gene have a critical role already in early stages of CRC development. The most important function of *APC* is to establish an interaction with the ß-catenin protein, thus accelerating its degradation and regulation of the cadherin-mediated cell–cell adhesion system. Functional loss of *APC* leads to abnormal ß-catenin accumulation and translocation from the plasma membrane to the nucleus [20].

Singh et al. used a whole exome sequencing approach and found *APC* mutations in 43% (3/7) of UrC samples. They detected one sample with a nonsense mutation, one with a frameshift mutation and one with a deletion. The frameshift mutation and deletion caused dysfunctional APC proteins [16]. Collazo-Lorduy et al. described truncated *APC* mutations (R1450*, R554*) in 22% (2/9) of UrC samples using a targeted exome sequencing approach [14]. In addition, Lee et al. reported *APC* mutations in 18% (3/17) of UrC cases including a frameshift deletion (K1444fs) and a stop-gain single nucleotide variant (SNV) (E1093*) [10]. In the latest study, Kardos et al. performed targeted exon sequencing of 12 urachal adenocarcinoma cases and found 3 of 12 samples (25%) with *APC* mutations [9].

In our study, analyzing 34 UrC cases, deleterious alterations of *APC* tumor suppressor gene were present in 2 UrC samples. Neither mutation has been previously reported in UrC: p.Y1075* (c.3225 T > G), p.K1199* (c.3595A > T). Both of these nonsense mutations are predicted to cause a truncated, dysfunctional APC protein. Three additional alterations were considered benign. Presence of potentially pathogenic *APC* mutations tended to be associated with shorter OS, however, because of the low number of cases with *APC* mutation, this correlation has to be interpreted cautiously.

Summarizing our results with all currently available data on *APC* status in UrC, an overall number of 14 of 141 (10%) UrC samples exhibited *APC* alterations [6–16, 18, 24–26] which is in clear contrast to the high *APC* mutational rate (80%) found in CRC [17]. This finding represents a further characteristic difference in the molecular taxonomy between CRC and UrC. Therefore, our results demonstrate that the Wnt pathway is probably less frequently involved in the pathogenesis of UrC compared to CRC.

ß-catenin is a well-established diagnostic and prognostic biomarker in CRC. Physiologically, ß-catenin staining is restricted to the membrane/cytoplasm and is involved in cadherin-mediated cell-cell adhesion and gene transcription regulation. Nuclear ß-catenin expression can be observed in CRC [19]. Wong et al. demonstrated positive nuclear staining for ß-catenin expression in the vast majority of colorectal adenocarcinomas which reflects the high *APC* mutation rate found in CRC [27].

In a recent review of the UrC literature including own data, we found a low incidence of nuclear ß-catenin staining in UrC (14%, 9/63) [4]. In the present study, we detected positive nuclear ß-catenin immunostaining in 29% (11/38) of UrC samples. In addition, ß-catenin nuclear expression was not associated with adverse OS (*p* = 0.606). Interestingly, we observed a nuclear ß-catenin localization only in one of the two samples with truncating APC mutation. As a potential genetic mechanism for ß-catenin nuclear accumulation, Alomar et al. identified an activating mutation in exon 3 of *CTNNB1* (ß-catenin) gene which resulted in an amino acid change at phosphorylation sites of glycogen synthase kinase-3 (GSK-3β). Failing of phosphorylation was found to decrease sequestration of β-catenin by *APC* [28]. This effect might explain our finding with low APC mutational frequency but at the same time surprising high rate (29%) of nuclear ß-catenin positivity. It has also to be kept in mind that we used a 1% threshold for calling a case positive in case of nuclear β-catenin staining. In some cases, it is difficult to discriminate between a real nuclear staining event in the background of strong membranous/cytoplasmic staining especially in smaller tumor cells (i.e. signet ring cells).

The tumor suppressor PTEN is a negative regulator of the PI3K signaling pathway. *PTEN* mutations occur in 4–10% of CRC and were suggested as potential markers of response to EGFR and mitogen-activated protein kinase (MAPK) inhibitor-based targeted therapies [17]. Perrone et al. showed that inactivation of PTEN protein by mutation (P103S, E99*) or deletion (hemizygous) was responsible of anti-EGFR resistance [29]. PTEN protein loss was detected in approximately 40% of all CRC patients, which is clearly higher compared to the rate of genomic loss. This suggests that PTEN is more frequently downregulated by epigenetic silencing. In accordance, *PTEN* methylation was found to be significantly correlated with PTEN expression [21]. Similar to the findings at the genomic level, Frattini et al. showed that loss of PTEN protein expression was associated with non-responsiveness to cetuximab [22].

In the literature, we found three whole exome sequencing studies with an overall number of 19 UrC samples. None of them reported any *PTEN* mutations [10, 14–16]. In addition, none of the targeted sequencing studies reported any *PTEN* mutations in UrC. Here, we identified a pathogenic frameshift *PTEN* mutation (p.C211fs) in one of the 34 UrC cancer samples (3%), suggesting a low incidence for *PTEN* mutation in UrC. Therefore, the *PTEN* mutational frequency in UrC seems to be similar to that of reported in CRC (4–10%) [17].

To the best of our knowledge, no published data is available on PTEN protein expression in UrC. In the present study, performing PTEN IHC analysis, we observed PTEN protein loss in 20% of UrC cases (6/30), which is somewhat lower than that of 30–40% described in CRC. The low rate of *PTEN* loss at the DNA level, but relative high rate at the protein level suggests that *PTEN* is predominantly silenced by epigenetic downregulation in UrC [21]. The high rate of PTEN protein loss in UrC suggests that its immunohistochemical analysis may be important in order to predict potential inresponsiveness to anti-EGFR therapy. In contrast, the rare occurrence of *PTEN* inactivating mutations suggests that these alterations are less important to analyze when considering the administration of an anti-EGFR drug, while activating *KRAS* mutations are more common in UrC (28%) and are clinically important negative predictors of anti-EGFR therapy [6–16, 18, 24–26].

## Conclusions

In summary, we analyzed a large cohort of UrC with a targeted next-generation sequencing approach supplemented with ß-catenin and PTEN immunohistochemical analyses. Our results show that *APC* mutations are much less frequent in UrC (10%) compared to CRC (80%) suggesting that the Wnt pathway is involved in the pathogenesis of only a relatively small portion of UrC. In addition, both UrC and CRC have low rates genomic loss (3% and 4–10%) but relative high rates of PTEN protein loss (20% and 40%), suggesting an epigenetic regulation for this gene in both tumor types. Therefore, as the loss of PTEN protein expression was found to be associated with resistance against anti-EGFR therapy, its immunohistochemical testing should be considered when planning anti-EGFR therapy. Our data provides further clues for both the similarity (regarding PTEN) and difference (regarding APC) between UrC and CRC and suggest that these tumor types are similar yet distinct on the molecular level.
